# Supplementary material for: Leveraging place field repetition to understand positional versus nonpositional inputs to hippocampal field CA1
Source: eLife. 2025 Apr 29;14:e85599. doi: 10.7554/eLife.85599 (PMC12040320; doi:10.7554/eLife.85599)
Supplement: Supplementary file 1. — However, major analyses were re-run with rewards included (i.e. all passes through the alley) to test whether this changed the results. In each of three major analyses, the inclusion of reward trials did not qualitatively change the results compared to when these trials were excluded. It should be noted for the re-analysis of Figure 3B, the numerator decreased by a large amount despite little change to the overall pool of fields tested. The fact that including rewards did not result in many more fields being included can be explained by the fact that rewards were uniformly distributed across the track so their removal decreased the samples from all fields evenly, resulting in little change to the number of fields included for analysis. The fact that the numerator (i.e. directional fields) decreased could be explained by the fact that rewarded trials introduced variability; indeed this was the justification for their removal, and this variability might have reduced the statistical power of the Mann-Whitney test from Figure 3C. [file elife-85599-supp1.docx]

**Supplementary Table 1. Inclusion of Rewarded Alley Traversals Did Not Affect Main Results**

| Analysis, figure | Result excluding rewards (from main text) | Result including rewards |
| --- | --- | --- |
| Proportion of fields with directional tuning (Fig 3B) | 93/310, binomial p = 3.6 x 10^-45^ | 73/313, binomial p = 2.08 x 10^-28^ |
| Lack of difference in directional tuning between repeating and nonrepeating neurons (Fig 4A) | Mann-Whitney U = 11883, p = 0.49 | Mann-Whitney U = 12067, p = 0.43 |
| Difference in correlation between directional tuning across fields in same versus different corridors (Fig 5E) | Real r^2^ = 0.22,  95^th^ percentile shuffle = 0.155 | Real r^2^ = 0.26,  95^th^ percentile shuffle = 0.156 |

Alley traversals on which the animal was rewarded were excluded from all main text analyses as any CA1 response to the reward itself was an undesirable source of variability. However, major analyses were re-run with rewards included (i.e. all passes through the alley) to test whether this changed the results. In each of three major analyses, the inclusion of reward trials did not qualitatively change the results compared to when these trials were excluded. It should be noted for the re-analysis of Fig 3B, the numerator decreased by a large amount despite little change to the overall pool of fields tested. The fact that including rewards did not result in many more fields being included can be explained by the fact that rewards were uniformly distributed across the track so their removal decreased the samples from all fields evenly, resuting in little change to the number of fields included for analysis. The fact that the numerator (i.e. directional fields) decreased could be explained by the fact that rewarded trials introduced variability; indeed this was the justification for their removal, and this variability might have reduced the statistical power of the Mann-Whitney test from Fig 3C.
