# Supplementary material for: Leveraging place field repetition to understand positional versus nonpositional inputs to hippocampal field CA1
Source: eLife. 2025 Apr 29;14:e85599. doi: 10.7554/eLife.85599 (PMC12040320; doi:10.7554/eLife.85599)
Supplement: MDAR checklist [file elife-85599-mdarchecklist1.docx]

**Materials Design Analysis Reporting (MDAR)**

**Checklist for Authors**

The [MDAR framework](https://osf.io/xfpn4/) establishes a minimum set of requirements in transparent reporting mainly applicable to studies in the life sciences.

*eLife* asks authors to **provide detailed information within their article** to facilitate the interpretation and replication of their work. Authors can also upload supporting materials to comply with relevant reporting guidelines for health-related research (see [EQUATOR Network](http://www.equator-network.org/%20)), life science research (see the [BioSharing Information Resource](http://biosharing.org/)), or animal research (see the [ARRIVE Guidelines](http://www.plosbiology.org/article/info:doi/10.1371/journal.pbio.1000412) and the [STRANGE Framework](https://doi.org/10.1038/d41586-020-01751-5); for details, see *eLife*’s [Journal Policies](https://reviewer.elifesciences.org/author-guide/journal-policies)). Where applicable, authors should refer to any relevant reporting standards materials in this form.

For all that apply, please note **where in the article** the information is provided. Please note that we also collect information about data availability and ethics in the submission form.

**Materials:**

| **Newly created materials** | **Indicate where provided: section/figure legend** | **N/A** |
| --- | --- | --- |
| The manuscript includes a dedicated "materials availability statement" providing transparent disclosure about availability of newly created materials including details on how materials can be accessed and describing any restrictions on access. |  | NA |
|  |  |  |
| **Antibodies** | **Indicate where provided: section/figure legend** | **N/A** |
| For commercial reagents, provide supplier name, catalogue number and [RRID](https://scicrunch.org/resources), if available. |  | NA |
|  |  |  |
| **DNA and RNA sequences** | **Indicate where provided: section/figure legend** | **N/A** |
| Short novel DNA or RNA including primers, probes: Sequences should be included or deposited in a public repository. |  | NA |
|  |  |  |
| **Cell materials** | **Indicate where provided: section/figure legend** | **N/A** |
| Cell lines: Provide species information, strain. Provide accession number in repository OR supplier name, catalog number, clone number, OR RRID. |  | NA |
| Primary cultures: Provide species, strain, sex of origin, genetic modification status. |  | NA |
|  |  |  |
| **Experimental animals** | **Indicate where provided: section/figure legend** | **N/A** |
| Laboratory animals or Model organisms: Provide species, strain, sex, age, genetic modification status. Provide accession number in repository OR supplier name, catalog number, clone number, OR RRID. | “*Animals and Pre-training” – wild type Long-Evans rats (*Rattus norvegicus) |  |
| Animal observed in or captured from the field: Provide species, sex, and age where possible. |  | NA |
|  |  |  |
| **Plants and microbes** | **Indicate where provided: section/figure legend** | **N/A** |
| Plants: provide species and strain, ecotype and cultivar where relevant, unique accession number if available, and source (including location for collected wild specimens). |  | NA |
| Microbes: provide species and strain, unique accession number if available, and source. |  | NA |
|  |  |  |
| **Human research participants** | **Indicate where provided: section/figure legend) or state if these demographics were not collected** | **N/A** |
| If collected and within the bounds of privacy constraints report on age, sex, gender and ethnicity for all study participants. |  | NA |

**Design:**

| **Study protocol** | **Indicate where provided: section/figure legend** | **N/A** |
| --- | --- | --- |
| If the study protocol has been pre-registered, provide DOI. For clinical trials, provide the trial registration number OR cite DOI. |  | NA |
|  |  |  |
| **Laboratory protocol** | **Indicate where provided: section/figure legend** | **N/A** |
| Provide DOI OR other citation details if detailed step-by-step protocols are available. |  | NA |
|  |  |  |
| **Experimental study design (statistics details) *** | | |
| **For in vivo studies: State whether and how the following have been done** | **Indicate where provided: section/figure legend. If it could have been done, but was not, write “not done”** | **N/A** |
| Sample size determination | Not done; sample size was decided based on best practices in the field and having observed the frequency with which place field repetition was observed in early animals. |  |
| Randomisation | Not done |  |
| Blinding | Not done |  |
| Inclusion/exclusion criteria | 1. “*Spike Sorting “* Cell isolation quality 2. “*Place Field Detection”* Place field size and overall firing rate 3. “*Data Filtering and Pre-processing “* Sampling requirements for field inclusion as well as putative interneuron exclusion 4. Manual exclusion based on rate maps and results of place field detection algorithm (Supplemental Figure 4) |  |
|  |  |  |
| **Sample definition and in-laboratory replication** | **Indicate where provided: section/figure legend** | **N/A** |
| State number of times the experiment was replicated in the laboratory. | >10 times and the 10 datasets with the best behavioral sampling and recording quality were used. |  |
| Define whether data describe technical or biological replicates. | 5 biological replicates (5 rats) and 2 technical replicates (2 best recording days per rat) for each biological replicate (i.e. 10 datasets, see above). |  |
|  |  |  |
| **Ethics** | **Indicate where provided: section/submission form** | **N/A** |
| Studies involving human participants: State details of authority granting ethics approval (IRB or equivalent committee(s), provide reference number for approval. |  | NA |
| Studies involving experimental animals: State details of authority granting ethics approval (IRB or equivalent committee(s), provide reference number for approval. | Materials & Methods. *Hyperdrive Construction and Implantation* . All animal procedures were approved by the Institutional Animal Care and Use Committee of Johns Hopkins University (Protocol number RA20A318) and complied with the Guide for the Care and Use of Laboratory Animals of the National Institutes of Health. |  |
| Studies involving specimen and field samples: State if relevant permits obtained, provide details of authority approving study; if none were required, explain why. |  | NA |
|  |  |  |
| **Dual Use Research of Concern (DURC)** | **Indicate where provided: section/submission form** | **N/A** |
| If study is subject to dual use research of concern regulations, state the authority granting approval and reference number for the regulatory approval. |  | NA |

**Analysis:**

| **Attrition** | **Indicate where provided: section/figure legend** | **N/A** |
| --- | --- | --- |
| Describe whether exclusion criteria were pre-established. Report if sample or data points were omitted from analysis. If yes, report if this was due to attrition or intentional exclusion and provide justification. | The categories of exclusion criteria were known in advance. For instance, cells with low isolation quality were removed as were passes through fields on which a reward was obtained (as that may have elicited a confounding response). Neurons thought to be interneurons (log mean rate > 0.6) were also excluded.  For some categories of exclusion criteria, the threshold of exclusion was determined after the experiment began, for instance the number of passes through each field needed for that field to be included (final value: 2passes).  Some neurons were also excluded based on their ratemaps after manual inspection. Cells without clear spatial firing fields, or firing that was insufficiently localized on the track were removed manually (Supplemental Figure 4) |  |
|  |  |  |
| **Statistics** | **Indicate where provided: section/figure legend** | **N/A** |
| Describe statistical tests used and justify choice of tests. | Tests are listed in order of appearance in the manuscript text. Where tests are performed multiple times, they are listed once with a justification common to all occurrences, e.g. using a t-test to compare sample means.   1. Chi-square goodness of fit (no figure) was used to compare frequencies of two groups. This is an appropriate test to determine whether counts from two groups are statistically equivalent or not. 2. Mann-Whitney U test was used to test difference between two population means in situations where assumptions of normality may not hold. 3. Orientation alignment score (OAS). This custom statistic was defined as the degree to which place fields shared a common orientation. The real value across the population was compared to a comparable value obtained through shuffling field orientation labels. This shuffling approach was justified as it controlled for the specific statistics of the data, for instance how many fields were horizontally- or vertically-oriented. 4. Binomial tests were used in conjunction with other statistical tests to determine if the number of data units (place fields, cells, etc) that passed a given test was more than expected by the false discovery rate. This is necessary because even under the null hypothesis some false positives will occur. 5. Generalized linear models were used to test whether certain variables, such as direction of travel or time, affected the place field response. GLMs are robust to non-normally distributed data and allow for the contributions of multiple variables to the firing response to be examined. 6. Likelihood ratio tests were used to compare GLM models to implement a hypothesis test where a variable of interest, e.g. direction, was added to a base model containing no such term. The LRT would test whether adding that variable significantly increased the explanatory power of the model and is a standard measure. 7. Random forest classifiers were used to test whether the population of place cells contained a directional signal, to complement the GLM/M-W tests done at the field level. This technique is a robust and widely-used technique in machine learning. The out-of-bag score is a standard method of assessing RF performance which is essentially a within-model cross-validation. To control for behavioral statistics that could (and did) affect the model, the OOB score was compared to a shuffled distribution. 8. A naïve classifier was used in conjunction with the random forest classifier. This classifier makes decisions solely based on behavior and its performance is a benchmark of to what extent behavioral confounds affected the real (RF) decoding performance. 9. Pearson’s Chi-square test was used to compare two different frequencies, expressed as ratios, to test whether one is larger. 10. The coefficient of determination (r^2^) was used to determine how well a linear regression model based on firing rates predicted position, i.e. the position decoding. The real r^2^ was compared to a shuffled distribution where position and spike timing were circularly shuffled with respect to one another. 11. A one-sample test (compared to 0) was used to determine if a group of numbers (the differences in repeating versus nonrepeating position decoding) were significantly different from 0, and this is a standard parametric way of addressing this question. 12. A two-way ANOVA (field identity x direction) was used to test whether there was an interaction at the cell-level between a specific place field of a repeating neuron and the field’s directionality. This is a standard use-case for the two-way ANOVA. 13. R^2^ was also used to determine how correlated two variables were, as is standard, throughout Figure 5. Here, a linear relationship between field directionality was used to test whether fields did or did not share their directional tuning. 14. A Fisher’s r-to-Z transform was used to compare two different correlations. This test is designed for that situation and was appropriate there. 15. A shuffling procedure was used to compare the real difference in r^2^ values for fields on the same or different corridors to a null distribution. This was done to control for the behavioral sampling statistics in the data. 16. Hartigan’s Dip statistic was used to test whether a distribution was unimodal or not. This test is appropriate as it tests for multiple peaks that would indicate non-unimodalness. 17. Pearson’s correlation was used to correlate similarities between response vectors. Specifically it was used to correlate the average response to shared paths between two fields (Fig 5G), the activity in different time windows (fig 6D) and to correlate the firing rate over time between different fields (Fig 6A). 18. The Spearman correlation was used to assess the change in position decoding performance before and after a training window. The Spearman correlation was used here because it evaluates a monotonic relationship in the response and here we were interested in the overall slope of the decoding performance. 19. (Supplement). Estimated marginalized means (emmeans) were used to test all pairwise comparisons of factor levels within a factor. This was done after fitting a GLM to the data and running emmeans on the directional regressors (previous, current, next). 20. (Supplement) Permutation shuffling tests were done as a control for the GLM effects. Supplemental Fig 10 controls for Fig 3C and Supplemental Fig 11 controls for Fig 6B. In both cases, labels corresponding to direction (Fig S10) or time (Fig S11) were shuffled 1000 times and the GLM procedure was repeated on the shuffled data. A shuffled distribution of the proportion of neurons with a (fictive) effect of the variable in question (direction, time) was created and the 95^th^ percentile of this shuffle was compared to the empirical result. |  |
|  |  |  |
| **Data availability** | **Indicate where provided: section/submission form** | **N/A** |
| For newly created and reused datasets, the manuscript includes a data availability statement that provides details for access (or notes restrictions on access). |  | NA |
| When newly created datasets are publicly available, provide accession number in repository OR DOI and licensing details where available. | The data are available at: https://drive.google.com/drive/folders/1W4BKht6AXN1RS5Xxz2u_ksZhX5ZgZUiY?usp=sharing |  |
| If reused data is publicly available provide accession number in repository OR DOI, OR URL, OR citation. |  | NA |
|  |  |  |
| **Code availability** | **Indicate where provided: section/figure legend** | **N/A** |
| For any computer code/software/mathematical algorithms essential for replicating the main findings of the study, whether newly generated or re-used, the manuscript includes a data availability statement that provides details for access or notes restrictions. |  | NA |
| Where newly generated code is publicly available, provide accession number in repository, OR DOI OR URL and licensing details where available. State any restrictions on code availability or accessibility. | https://github.com/whock3/ratterdam/tree/eLife_repetition_manuscript_code/RatterdamOpen_Project/repetition_manuscript_code |  |
| If reused code is publicly available provide accession number in repository OR DOI OR URL, OR citation. |  | NA |

**Reporting:**

The MDAR framework recommends adoption of discipline-specific guidelines, established and endorsed through community initiatives.

| **Adherence to community standards** | **Indicate where provided: section/figure legend** | **N/A** |
| --- | --- | --- |
| State if relevant guidelines (e.g., ICMJE, MIBBI, ARRIVE, STRANGE) have been followed, and whether a checklist (e.g., CONSORT, PRISMA, ARRIVE) is provided with the manuscript. |  | NA |

* We provide the following guidance regarding transparent reporting and statistics; we also refer authors to [Ten common statistical mistakes to watch out for when writing or reviewing a manuscript](https://doi.org/10.7554/eLife.48175).

**Sample-size estimation**

- You should state whether an appropriate sample size was computed when the study was being designed
- You should state the statistical method of sample size computation and any required assumptions
- If no explicit power analysis was used, you should describe how you decided what sample (replicate) size (number) to use

**Replicates**

- You should report how often each experiment was performed
- You should include a definition of biological versus technical replication
- The data obtained should be provided and sufficient information should be provided to indicate the number of independent biological and/or technical replicates
- If you encountered any outliers, you should describe how these were handled
- Criteria for exclusion/inclusion of data should be clearly stated
- High-throughput sequence data should be uploaded before submission, with a private link for reviewers provided (these are available from both GEO and ArrayExpress)

**Statistical reporting**

- Statistical analysis methods should be described and justified
- Raw data should be presented in figures whenever informative to do so (typically when N per group is less than 10)
- For each experiment, you should identify the statistical tests used, exact values of N, definitions of center, methods of multiple test correction, and dispersion and precision measures (e.g., mean, median, SD, SEM, confidence intervals; and, for the major substantive results, a measure of effect size (e.g., Pearson's r, Cohen's d)
- Report exact p-values wherever possible alongside the summary statistics and 95% confidence intervals. These should be reported for all key questions and not only when the p-value is less than 0.05.

**Group allocation**

- Indicate how samples were allocated into experimental groups (in the case of clinical studies, please specify allocation to treatment method); if randomization was used, please also state if restricted randomization was applied
- Indicate if masking was used during group allocation, data collection and/or data analysis
